# Hypoxia-Inducible Factor-Prolyl-Hydroxylase and Sodium-Glucose Cotransporter 2 Inhibitors for Low-Risk Myelodysplastic Syndrome-Related Anemia in Patients with Chronic Kidney Disease: A Report of Three Cases

**DOI:** 10.3390/hematolrep15010019

**Published:** 2023-03-06

**Authors:** Satoshi Yamasaki, Takahiko Horiuchi

**Affiliations:** 1 Department of Internal Medicine, Kyushu University Beppu Hospital, Beppu 874-0840, Japan; 2 Department of Hematology and Clinical Research Institute, National Hospital Organization Kyushu Medical Center, Fukuoka 810-0065, Japan

**Keywords:** myelodysplastic syndrome, anemia, chronic kidney disease, hypoxia-inducible factor-prolyl-hydroxylase inhibitor, sodium-glucose cotransporter 2 inhibitor

## Abstract

Although daprodustat, a hypoxia-inducible factor prolyl hydroxylase inhibitor, and dapagliflozin, a sodium-glucose cotransporter 2 inhibitor, have been approved for the treatment of renal anemia in Japan, their efficacy and safety for patients aged 80 years or older with low-risk myelodysplastic syndrome (MDS)-related anemia have not been demonstrated. Our case series comprised two men and one woman aged >80 years with low-risk MDS-related anemia and diabetic mellitus (DM)-related chronic kidney disease who were dependent on red blood cell transfusions and in whom erythropoiesis-stimulating agents had been insufficient. All three patients received daprodustat and additional dapagliflozin achieved red blood cell transfusion independence and were followed up for >6 months. Daily oral daprodustat was well tolerated. There were no fatalities or progression to acute myeloid leukemia during the >6-month follow-up after daprodustat initiation. On the basis of these outcomes, we consider 24 mg of daprodustat combined with 10 mg of dapagliflozin daily an effective form of treatment for low-risk MDS-related anemia. Further studies are required to clarify the synergistic effects of daprodustat and dapagliflozin, which correct chronic kidney disease-related anemia by promoting endogenous erythropoietin production and normalizing iron metabolism to manage low-risk MDS in the long term.

## 1. Background

Myelodysplastic syndrome (MDS) is a general term for a heterogeneous group of diseases characterized by ineffective hematopoiesis and cytopenia [1]. Several scoring systems are available to evaluate the prognosis of patients with MDS, and the most commonly used systems are the International Prognostic Scoring System (IPSS) [2], revised-IPSS [3], and World Health Organization (WHO) classification-based system [4]. MDS treatment is determined in accordance with the patient’s risk classification. Approximately 77% of patients with MDS have low-risk MDS (LR-MDS) at the time of diagnosis, as defined by a revised-IPSS score of ≤3.5 [5]. LR-MDS treatment goals are to improve cytopenia, reduce the need for red blood cell (RBC) transfusion, improve quality of life, prolong overall survival, and if possible, reduce the risk of progression to leukemia [1]. Although it occurs in almost all age groups, MDS mostly affects older persons, posing important problems for hematologists, especially concerning diagnosis and the ability to determine and administer appropriate treatment in a timely manner [1]. Over 90% of patients diagnosed with MDS have anemia at the time of diagnosis, and over 60% of them develop severe anemia at later stages of their disease [6,7]. A few treatments for anemia with variable response rates have recently been approved for patients with LR-MDS [1]. Erythropoiesis-stimulating agents (ESAs) are approved in Japan for patients with LR-MDS with symptomatic anemia, hemoglobin <10 g/dL, and serum erythropoietin (EPO) concentrations <500 mU/mL [8]. RBC transfusions are also used to treat LR-MDS-induced anemia. However, frequent RBC transfusions have clinically and economically harmful effects [9]. Although ESAs are generally effective, they are inconvenient because they are administered subcutaneously and have highly variable erythroid response rates, and the response capacity of patients with chronic kidney disease (CKD) varies widely [10].

Daprodustat is an orally administered, hypoxia-inducible factor (HIF) prolyl hydroxylase (PHD) inhibitor that corrects anemia by a mechanism of action that is different from that of ESAs [11]. Daprodustat stimulates erythropoiesis by inhibiting the HIF-PHD enzymes PHD1, PHD2, and PHD3. This leads to the stabilization of HIF-α transcription factors and induction of HIF-responsive genes involved in adaptation to hypoxia including EPO, the endothelial growth factor, and genes that regulate iron uptake, mobilization, and transport, resulting in decreased hepcidin production [12]. This corrects chronic kidney disease (CKD)-related anemia. Daprodustat was approved in Japan to treat chronic kidney disease CKD-related amenia in August 2020 but not MDS-related anemia. Daprodustat is currently being evaluated as an oral alternative to conventional ESA therapy. On the basis of its effectiveness in treating CKD-related anemia that occurs because of low EPO levels, we hypothesized that daprodustat might achieve clinical benefit in LR-MDS-related anemia multifactorial, in which dyserythropoesis in the bone marrow niche is the predominant pathology.

Pivotal and novel mechanisms for glomerular hyperfiltration, renal anemia, hypoxia, and energy imbalance are emerging for diabetic mellitus (DM)-related CKD. Additionally, HIF stabilizer and sodium-glucose cotransporter 2 inhibitors (SGLT2is) [13] are thought to have resulted in paradigm shifts in the treatment and prevention of DM-related CKD. SGLT2is that were used to treat albuminuria reportedly achieved better pre-specified renal outcomes, including the glomerular filtration rate, in studies such as the cardiovascular outcome trials (empagliflozin for EMPA-REG [14], canagliflozin for CANVAS [15], and dapagliflozin for DECLARE-TIMI [16]). Dapagliflozin was approved in Japan to treat DM-related CKD in August 2021, but it is not approved to treat MDS-related anemia.

Currently, treatment of LR-MDS patients focuses on combating cytopenia, especially anemia, and the poor consequences of transfusion load. However, there are still unmet needs regarding the treatment of LR-MDS-related anemia in patients with DM-related CKD. The primary goal of this case series was to investigate the synergistic effects of daprodustat and dapagliflozin, which corrects CKD-related anemia by promoting endogenous erythropoietin production and normalizing iron metabolism in LR-MDS patients with DM-related CKD in the real world.

## 2. Case Presentation

### 2.1. Case 1

**Figure 1:**
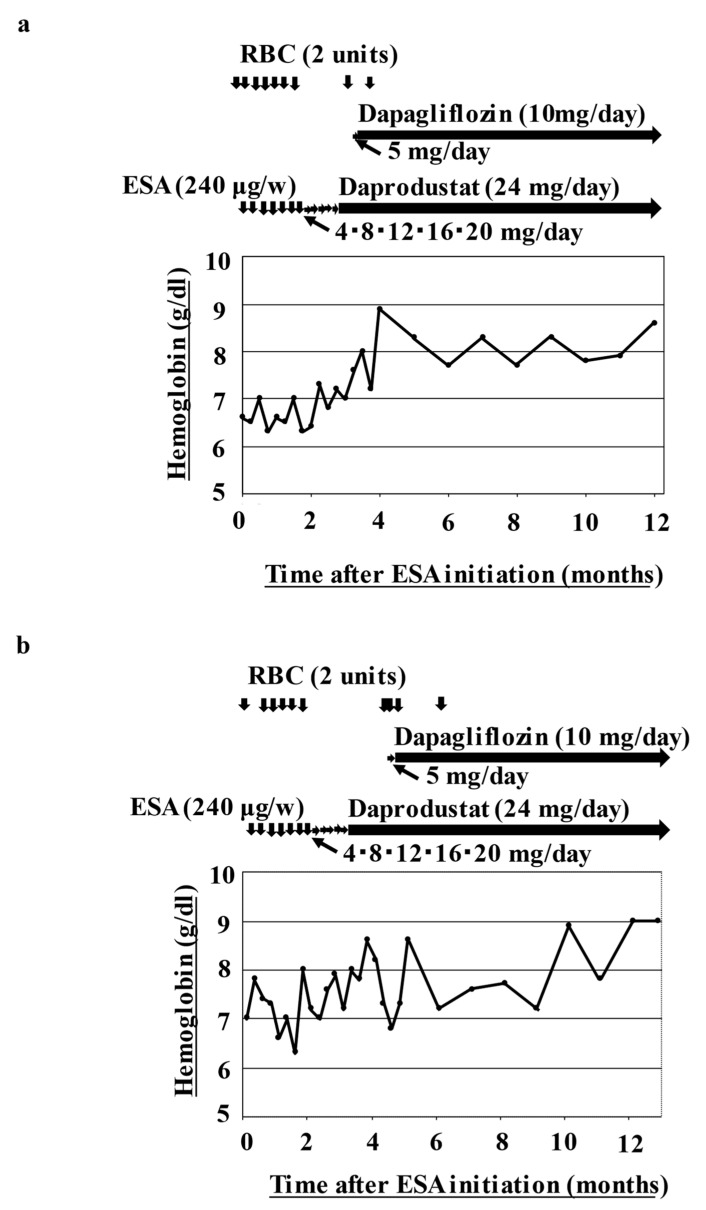

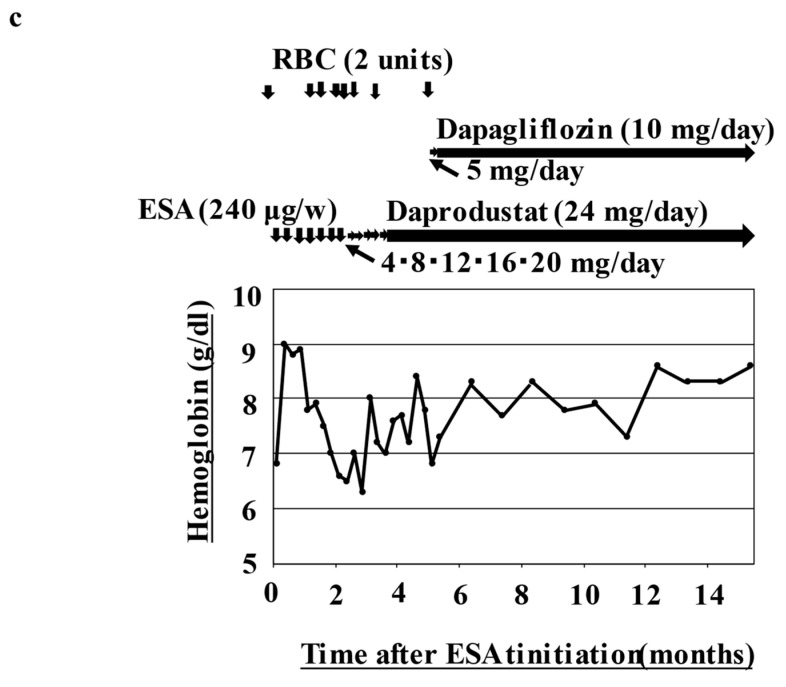
Changes in hemoglobin concentrations and treatment administered. After initiation of erythropoiesis-stimulating agents, the patients’ hemoglobin concentrations began to improve. The upper panels show the frequency of red blood cell transfusion and treatments administered, while the lower panels show the hemoglobin concentrations ((**a**), case 1; (**b**), case 2; (**c**), case 3). ESA, erythropoiesis-stimulating agent; RBC, red blood cell; w, week.

An 80-year-old man was diagnosed with MDS by bone marrow aspiration in accordance with the WHO 2016 guidelines. His baseline clinical and biological characteristics are presented in Table 1. He had refractory anemia, as defined by the WHO 2016 guidelines, and his disease was categorized as low risk in accordance with the revised IPSS (IPSS-R Int-1). DM was diagnosed 26 years previously and had resulted in renal insufficiency for which he had not undergone hemodialysis, but he had been treated with oral metformin and dipeptidyl-peptidase-4 (DPP-4) inhibitors. Weekly ESA treatment was started when he became RBC transfusion-dependent, with hemoglobin concentrations maintained at >7.0 g/dL. However, even after starting ESA treatment, he continued to require RBC transfusions. These frequent RBC transfusions caused congestive heart failure and secondary hemochromatosis, resulting in a diagnosis of RBC transfusion-associated circulatory overload, and thus, oral daprodustat (up to 24 mg/day) was substituted for the ESA 3 years after the MDS diagnosis. After starting daprodustat, the anemia resolved. However, 7 weeks after starting daprodustat, he again required RBC transfusions and was accordingly started on 5–10 mg of oral dapagliflozin daily. No RBC transfusions were required for the next 6 months, and his hemoglobin concentration was maintained at >7 g/dL (Figure 1a). Throughout this period, no physical changes or particular adverse events, including thrombosis and infections, were noted. Thus, we concluded that the decrease in hemoglobin was attributable to the failure of ESA treatment and that daprodustat combined with dapagliflozin had increased his hemoglobin concentrations.

### 2.2. Case 2

A 93-year-old woman was diagnosed with low-risk MDS (IPSS-R low) with refractory anemia in accordance with the WHO 2016 guidelines. She was diagnosed with DM 23 years previously, and it was associated CKD that did not require hemodialysis. Her DM was treated with oral metformin and DPP-4 inhibitors. When she became RBC transfusion-dependent, weekly ESA treatments started. However, she continued to require RBC transfusions. Four years later, oral daprodustat at up to 24 mg/day was substituted for the ESA. After starting daprodustat, her anemia resolved. However, after 8 weeks of daprodustat treatment, she again required RBC transfusions and was started on 5–10 mg of oral dapagliflozin daily. She required no RBC transfusions for the next 8 months because her hemoglobin concentration was maintained at >7 g/dL (Figure 1b).

### 2.3. Case 3

An 81-year-old man with essential thrombocythemia gradually developed anemia and was diagnosed with therapy-related, low-risk MDS (IPSS-R low) with refractory anemia 4 years later in accordance with the WHO 2016 guidelines. He was diagnosed with DM 21 years previously, and it was associated with CKD which did not require hemodialysis. His DM had been treated with oral metformin and DPP-4 inhibitors. When he became RBC transfusion-dependent, weekly ESA treatments started. The anemia temporarily improved with ESAs, but the patient again became RBC transfusion-dependent. Thus, oral daprodustat (up to 24 mg/day) was substituted for the ESA. After starting daprodustat treatment, the anemia resolved. However, after 7 weeks of daprodustat treatment, he again required RBC transfusions and was started on 5–10 mg of oral dapagliflozin daily. He needed no RBC transfusions for the next 9 months because his hemoglobin concentration was maintained at >7 g/dL (Figure 1c).

## 3. Discussion

We found that 24 mg of daprodustat combined with 10 mg of dapagliflozin might be effective against LR-MDS-related anemia in patients aged 80 years and older with DM-related CKD who were transfusion dependent and in whom ESAs had failed, but prospective randomized controlled trials using 24 mg of daprodustat combined with 10 mg of dapagliflozin to treat LR-MDS-related anemia are required to further investigate this regimen.

In a normoxic environment, hypoxia-inducible factor-prolyl-hydroxylase (HIF-PH) targets HIF-α for degradation through hydroxylation. However, under hypoxic conditions, HIF-PH is unable to hydroxylate HIF-α, which allows it to translocate to the cell nucleus where it dimerizes with HIF-β to form a functional HIF transcription factor and promote transcription of several genes, including those involved in EPO production [17]. Daprodustat mimics the body’s natural response to hypoxia by inhibiting HIF-PH, preventing hydroxylation of HIF-α, and allowing for the transcription and expression of genes that are necessary for erythropoiesis, particularly EPO and factors involved in iron metabolism [18]. HIF-PH improves renal anemia by promoting endogenous EPO production and normalizing iron metabolism. HIF-PH inhibitors may be useful for treating patients with MDS, but their efficacy and safety have been studied currently in the ongoing double-blind study phase and the trial remains blinded, so the data are not available to analyze at this time [19]. HIF-PH inhibitors have recently been developed as a new treatment for CKD-related anemia. Anemia is a common and major complication in CKD patients and characteristically progresses as renal function deteriorates. The safety and efficacy of daprodustat compared with those of darbepoetin were evaluated in a trial of 3872 patients with nondialysis CKD and anemia who were randomly assigned to receive daprodustat or darbepoetin. Patients were followed for a median of approximately 2 years [11]. Although hemoglobin concentrations increased more with daprodustat therapy, cardiovascular events, such as a composite of death, nonfatal stroke, and nonfatal myocardial infarction were more frequent with daprodustat.

Several recent studies have reported that SGLT2is have renal benefits in addition to glucose control [20]. The renal benefits of SGLT2is can be linked to the alleviation of renal function-related anemia [21]. Renal anemia may develop earlier and be worse in patients with DM-related CKD than in those with non-DM-related CKD [22]. Generally, all risk factors for renal anemia in the general population may also cause anemia in patients with DM-related CKD. These risk factors include aging kidneys, deficiency of factors required for RBC production, blood loss including bleeding tendencies caused by uremic or antiplatelet-related coagulopathy, and advanced glycation end production-related RBC deformability [23]. Additionally, SGLT2is, including dapagliflozin, were originally developed to treat DM, but off-target effects of SGLT2is in both renal and heart failure have attracted attention. Dapagliflozin is not recommended for use in patients with active bladder cancer [24], and there is limited experience in patients with severe hepatic impairment. The most common side effects of SGLT2is are vulvovaginal candidal infections and hypotension, acute kidney injury, urinary tract infections, necrotizing fasciitis of the perineum, euglycemic diabetic ketoacidosis, increased risk of lower extremity amputation, and bone fractures have also been reported [24].

This case series had several limitations, including that it was a small, single-institute series. Objective data, such as laboratory values, are reliable. However, subjective data such as non-hematological toxicity may have been underestimated because this information was dependent on medical records written by physicians before the study was planned. For insurance requirements, we included only patients with low-risk MDS who had DM-related CKD and were RBC transfusion-dependent, and in whom ESAs had failed because daprodustat for renal anemia and dapagliflozin for DM or CKD is covered by insurance in Japan. We explicitly set these criteria to minimize the underestimation of any correlations with adverse effects and drug interactions that resulted from selection bias. However, there is no way to know if the improvement in the patients’ anemia was due to the management of the underlying MDS or CKD.

In summary, our findings suggest that treatment with daprodustat combined with dapagliflozin might be of benefit in patients aged 80 years or older with MDS-related anemia and DM-related CKD who were transfusion-dependent and in whom ESA had failed. Possible mechanisms of the synergistic effects of daprodustat and dapagliflozin may depend on the decreased hepcidin production, which corrects CKD-related anemia mediated by daprodustat and the off-target effects of dapagliflozin in both renal and heart failure, which improves CKD-related anemia by promoting endogenous EPO production and normalizing iron metabolism. Additional long-term studies and prospective randomized controlled trials on 24 mg of daprodustat combined with 10 mg of dapagliflozin to treat LR-MDS-related anemia are required to clarify the role of daprodustat combined with dapagliflozin in managing MDS.

## Figures and Tables

**Table 1:** Baseline clinical and biologic characteristics.

Features	Case 1	Case 2	Case 3
Age, years at diagnosis of MDS	80	93	81
Sex	Male	Female	Male
MDS duration, years	3	4	5
WHO classification of MDS	MLD	SLD	SLD
Cytogenetic actual category at diagnosis	del(20q)	Normal	-Y
Bone marrow blasts at diagnosis, %	2.1	1.4	1.6
Bone marrow blasts post-dapagliflozin, %	1.8	1.2	2.0
ECOG performance status prior to ESAs	1	2	1
Serum EPO level prior to ESAs, mIU/mL	486	165	492
pRBC/8-weeks over 16 consecutive weeks prior to ESAs	4	2	8
Ferritin prior to ESAs, ng/mL	1472	1401	1224
eGFR prior to ESAs, mL/min	32.3	20.6	32.1
eGFR prior to dapagliflozin, mL/min	22.3	19.9	27.2
eGFR post-dapagliflozin, mL/min	25.0	22.3	32.9
Glycoalbumin prior to dapagliflozin, %	17.8	17.7	17.0
Glycoalbumin post-dapagliflozin, %	17.3	16.8	16.5
Reticulocyte counts prior to dapagliflozin, %	1.9	1.8	1.5
Reticulocyte counts post-dapagliflozin, %	2.5	3.0	2.6

ECOG, Eastern Cooperative Oncology Group; eGRF, estimated glomerular filtration; EPO, erythropoietin; ESAs, erythropoiesis-stimulating agents; MDS-MLD, MDS with multilineage dysplasia; MDS-SLD, MDS with single lineage dysplasia; MDS, myelodysplastic syndrome; pRBC, packed red blood cells; WHO, World Health Organization.

## Data Availability

The data that support the findings of this study are available upon request from the corresponding author. The data are not publicly available because of privacy or ethical restrictions.

## References

[1] Steensma D.P. (2018). Myelodysplastic syndromes current treatment algorithm 2018. Blood Cancer J..

[2] Greenberg P., Cox C., LeBeau M.M., Fenaux P., Morel P., Sanz G., Sanz M., Vallespi T., Hamblin T., Oscier D. (1997). International scoring system for evaluating prognosis in myelodysplastic syndromes. Blood.

[3] Greenberg P.L., Tuechler H., Schanz J., Sanz G., Garcia-Manero G., Solé F., Bennett J.M., Bowen D., Fenaux P., Dreyfus F. (2012). Revised international prognostic scoring system for myelodysplastic syndromes. Blood.

[4] Malcovati L., Della Porta M.G., Strupp C., Ambaglio I., Kuendgen A., Nachtkamp K., Travaglino E., Invernizzi R., Pascutto C., Lazzarino M. (2011). Impact of the degree of anemia on the outcome of patients with myelodysplastic syndrome and its integration into the WHO classification-based prognostic scoring system (WPSS). Haematologica.

[5] Arber D.A., Orazi A., Hasserjian R., Thiele J., Borowitz M.J., Le Beau M.M., Bloomfield C.D., Cazzola M., Vardiman J.W. (2016). The 2016 revision to the World health organization classification of myeloid neoplasms and acute leukemia. Blood.

[6] Haferlach T., Nagata Y., Grossmann V., Okuno Y., Bacher U., Nagae G., Schnittger S., Sanada M., Kon A., Alpermann T. (2014). Landscape of genetic lesions in 944 patients with myelodysplastic syndromes. Leukemia.

[7] Trowbridge J.J., Starczynowski D.T. (2021). Innate immune pathways and inflammation in hematopoietic aging, clonal hematopoiesis, and MDS. J. Exp. Med..

[8] Kubasch A.S., Platzbecker U. (2019). Setting fire to ESA and EMA resistance: New targeted treatment options in lower risk myelodysplastic syndromes. Int. J. Mol. Sci..

[9] Malcovati L., Germing U., Kuendgen A., Della Porta M.G., Pascutto C., Invernizzi R., Giagounidis A., Hildebrandt B., Bernasconi P., Knipp S. (2007). Time-dependent prognostic scoring system for predicting survival and leukemic evolution in myelodysplastic syndromes. J. Clin. Oncol..

[10] Gillespie I.A., Macdougall I.C., Richards S., Jones V., Marcelli D., Froissart M. (2015). Factors precipitating erythropoiesis-stimulating agent responsiveness in a European haemodialysis cohort: Case-crossover study. Pharmacoepidemiol Drug. Saf..

[11] Singh A.K., Carroll K., McMurray J.J.V., Solomon S., Jha V., Johansen K.L., Lopes R.D., Macdougall I.C., Obrador G.T., Waikar S.S. (2021). Daprodustat for the treatment of Anemia in patients not undergoing dialysis. N. Engl. J. Med..

[12] Liu Q., Davidoff O., Niss K., Haase V.H. (2012). Hypoxia-inducible factor regulates hepcidin via erythropoietin-induced erythropoiesis. J. Clin. Invest..

[13] Kanbay M., Tapoi L., Ureche C., Tanriover C., Cevik E., Afsar B., Cherney D.Z.I., Covic A. (2021). Effect of sodium-glucose cotransporter 2 inhibitors on hemoglobin and hematocrit levels in type 2 diabetes: A systematic review and meta-analysis. Int. Urol. Nephrol..

[14] Wanner C., Inzucchi S.E., Lachin J.M., Fitchett D., von Eynatten M., Mattheus M., Johansen O.E., Woerle H.J., Broedl U.C., Zinman B. (2016). Empagliflozin and progression of kidney disease in type 2 diabetes. N. Engl. J. Med..

[15] Neal B., Perkovic V., Mahaffey K.W., de Zeeuw D., Fulcher G., Erondu N., Shaw W., Law G., Desai M., Matthews D.R. (2017). Canagliflozin and cardiovascular and renal events in type 2 diabetes. N. Engl. J. Med..

[16] Mosenzon O., Wiviott S.D., Cahn A., Rozenberg A., Yanuv I., Goodrich E.L., Murphy S.A., Heerspink H.J.L., Zelniker T.A., Dwyer J.P. (2019). Effects of dapagliflozin on development and progression of kidney disease in patients with type 2 diabetes: An analysis from the DECLARE-TIMI 58 randomised trial. Lancet Diabetes Endocrinol..

[17] Greer S.N., Metcalf J.L., Wang Y., Ohh M. (2012). The updated biology of hypoxia-inducible factor. EMBO J..

[18] Ariazi J.L., Duffy K.J., Adams D.F., Fitch D.M., Luo L., Pappalardi M. (2017). Discovery and Preclinical characterization of GSK1278863 (Daprodustat), a small molecule hypoxia inducible factor-prolyl hydroxylase inhibitor for anemia. J. Pharmacol. Exp. Ther..

[19] Henry D.H., Glaspy J., Harrup R., Mittelman M., Zhou A., Carraway H.E. (2022). Roxadustat for the treatment of anemia in patients with lower-risk myelodysplastic syndrome: Open-label, dose-selection, lead-in stage of a phase 3 study. Am. J. Hematol..

[20] Toyama T., Neuen B.L., Jun M., Ohkuma T., Neal B., Jardine M.J. (2019). Effect of SGLT2 inhibitors on cardiovascular, renal and safety outcomes in patients with type 2 diabetes mellitus and chronic kidney disease: A systematic review and meta-analysis. Diabetes Obes. Metab..

[21] Mende C.W. (2022). Chronic kidney disease and SGLT2 inhibitors: A review of the evolving treatment landscape. Adv. Ther..

[22] Mehdi U., Toto R.D. (2009). Anemia, diabetes, and chronic kidney disease. Diabetes Care..

[23] Yamazaki T., Mimura I., Tanaka T., Nangaku M. (2021). Treatment of diabetic kidney disease: Current and future. Diabetes Metab. J..

[24] Kurata Y., Nangaku M. (2022). Dapagliflozin for the treatment of chronic kidney disease. Expert. Rev. Endocrinol. Metab..

